# Combined Effects of Ketogenic Diet and Aerobic Exercise on Skeletal Muscle Fiber Remodeling and Metabolic Adaptation in Simulated Microgravity Mice

**DOI:** 10.3390/metabo15040270

**Published:** 2025-04-13

**Authors:** Jun Chen, Wenjiong Li, Liang Yu, Bowei Zhang, Zhili Li, Peng Zou, Bai Ding, Xiaoqian Dai, Qirong Wang

**Affiliations:** 1 School of Exercise and Health, Shanghai University of Sport, Shanghai 200438, China; junchen_chn@163.com; 2 Sports Nutrition Center, National Institute of Sports Medicine, Beijing 100029, China; 3 National Key Laboratory of Space Medicine, Beijing 100094, China; muzijiong2007@163.com (W.L.); li_zhili777@163.com (Z.L.); zoup005@163.com (P.Z.); dingbai2001@aliyun.com (B.D.); 4 Department of Exercise Physiology, Beijing Sport University, Beijing 100084, China; yuliang@bsu.edu.cn (L.Y.); a18811300973@163.com (B.Z.)

**Keywords:** ketogenic diet, aerobic exercise, muscle fibers, exercise capacity

## Abstract

**Objective:** Prolonged microgravity environments impair skeletal muscle homeostasis by triggering fiber-type transitions and metabolic dysregulation. Although exercise and nutritional interventions may alleviate disuse atrophy, their synergistic effects under microgravity conditions remain poorly characterized. This study investigated the effects of an 8-week ketogenic diet combined with aerobic exercise in hindlimb-unloaded mice on muscle fiber remodeling and metabolic adaptation. **Methods:** Seven-week-old male C57BL/6J mice were randomly divided into six groups: normal diet control (NC), normal diet with hindlimb unloading (NH), normal diet with hindlimb unloading and exercise (NHE), ketogenic diet control (KC), ketogenic diet with hindlimb unloading (KH), and ketogenic diet with hindlimb unloading and exercise (KHE). During the last two weeks of intervention, hindlimb unloading was applied to simulate microgravity. Aerobic exercise groups performed moderate-intensity treadmill running (12 m/min, 60 min/day, and 6 days/week) for 8 weeks. Body weight, blood ketone, and glucose levels were measured weekly. Post-intervention assessments included the respiratory exchange ratio (RER), exhaustive exercise performance tests, and biochemical analyses of blood metabolic parameters. The skeletal muscle fiber-type composition was evaluated via immunofluorescence staining, lipid deposition was assessed using Oil Red O staining, glycogen content was analyzed by Periodic Acid–Schiff (PAS) staining, and gene expression was quantified using quantitative real-time PCR (RT-qPCR). **Results:** Hindlimb unloading significantly decreased body weight, induced muscle atrophy, and reduced exercise endurance in mice. However, the combination of KD and aerobic exercise significantly attenuated these adverse effects, as evidenced by increased proportions of oxidative muscle fibers (MyHC-I) and decreased proportions of glycolytic fibers (MyHC-IIb). Additionally, this combined intervention upregulated the expression of lipid metabolism-associated genes, including CPT-1b, HADH, PGC-1α, and FGF21, enhancing lipid metabolism and ketone utilization. These metabolic adaptations corresponded with improved exercise performance, demonstrated by the increased time to exhaustion in the KHE group compared to other hindlimb unloading groups. **Conclusions:** The combination of a ketogenic diet and aerobic exercise effectively ameliorates simulated microgravity-induced skeletal muscle atrophy and endurance impairment, primarily by promoting a fiber-type transition from MyHC-IIb to MyHC-I and enhancing lipid metabolism gene expression (CPT-1b, HADH, and PGC-1α). These findings underscore the potential therapeutic value of combined dietary and exercise interventions for mitigating muscle atrophy under simulated microgravity conditions.

## 1. Introduction

In recent years, advancements in space exploration and long-term microgravity research have increasingly drawn the attention of sports scientists toward the effects of weightlessness on muscle health. Numerous studies indicate that microgravity conditions can cause significant structural and functional alterations in skeletal muscles, particularly in antigravity muscles, including muscle atrophy, changes in muscle fiber types, and reduced motor function [1], subsequently impairing overall health. Consequently, developing effective interventions to mitigate or reverse the adverse effects of microgravity on muscles has become a critical research area in sports science.

The ketogenic diet (KD), characterized by high fat, moderate protein, and very low carbohydrate intake [2,3], stimulates ketone body production—primarily, β-hydroxybutyrate (β-HB) and acetoacetate—as alternative energy substrates [4]. These metabolites enhance muscular endurance and strength by providing efficient energy sources for skeletal muscles, thereby potentially improving athletic performance [5,6]. Emerging evidence suggests that KD may further support muscular adaptive changes by promoting muscle tissue remodeling under metabolic stress, including shifts in the muscle fiber-type composition, which is critical for optimizing endurance and muscular function [7]. For instance, studies on aged mice have demonstrated that KD interventions increase skeletal muscle mass and enhance functional capacity. Specifically, prolonged KD reduces the cross-sectional area of Myosin Heavy Chain (MyHC)-IIb fibers while increasing the area of MyHC-IIa fibers in senescent muscles [8], highlighting its potential in mitigating age-related muscle disorders, such as sarcopenia. These findings align with prior observations that KD preserves muscle mass in sedentary animals and facilitates MyHC isoform transitions from type IIb to IIx [9]. Conversely, reduced skeletal muscle activity triggers metabolic remodeling, characterized by decreased fat oxidation capacity, enhanced glycolytic metabolism, and a slow-to-fast twitch myosin isoform transition [10,11]. Notably, aerobic exercise, a conventional intervention against sarcopenia, has also been shown to stimulate ketogenesis [12], suggesting potential synergy when combined with KD. Animal studies further report enhanced endurance performance following KD regimens lasting 5 [13], 8 [14], and 12 [15] weeks. Previous research has also indicated that combining aerobic exercise with KD preferentially enhances fatty acid utilization and lipid oxidation without negatively affecting muscle glucose utilization or glycogen content [16], holding promise for optimizing lipid metabolism to improve endurance performance. Although prior studies highlight that either exercise or nutritional interventions alone can counteract muscle atrophy across various pathophysiological conditions [17,18,19], the combined effects of KD and aerobic exercise on microgravity-induced muscular atrophy remain underexplored.

Based on existing evidence, we hypothesize that combining KD with aerobic exercise under simulated microgravity conditions will synergistically enhance skeletal muscle mass and exercise capacity in mice. We propose that this combined intervention modulates the muscle fiber-type composition and metabolic adaptations, thus delaying or preventing muscle atrophy progression. This study aims to elucidate the integrated effects of KD and aerobic exercise on skeletal muscle adaptability, providing novel insights for aerospace medicine, sports science, and nutritional interventions. Using 7-week-old C57BL/6J mice, this study investigates how an 8-week KD combined with aerobic exercise influences skeletal muscle fiber characteristics and exercise performance in simulated microgravity, aiming to establish foundational insights for targeted interventions against microgravity-induced muscle atrophy.

## 2. Materials and Methods

### 2.1. Animals and Diets

Seven-week-old male C57BL/6J mice (20–25 g) were purchased from Beijing Vital River Laboratory Animal Technology Co., Ltd. (Beijing, China) and housed under standard laboratory conditions. Mice were housed in cages containing 4–5 animals each and maintained in a controlled environment with a temperature of 20–24 °C, relative humidity of 45–55%, and a 12 h light/dark cycle. All experimental procedures were approved by the Ethics Committee of the National Key Laboratory of Space Medicine (approval no.: ACC-IACUC-2021-010). Following a one-week acclimation period, mice were stratified by body weight and randomly assigned to six experimental groups using SPSS 22.0 (IBM, Armonk, NY, USA) to ensure a balanced allocation. The experimental design comprised two dietary conditions (normal diet (ND) and ketogenic diet (KD)) and three intervention types: control (C), hindlimb unloading (HU), and hindlimb unloading combined with aerobic exercise (HU + EX). Sample sizes were determined as follows: the control groups (NC and KC) contained 8 mice each, while the HU and HU + EX groups (NH, NHE, KH, and KHE) were expanded to 12–14 mice to account for potential attrition during prolonged interventions. The detailed group allocation is presented in Table 1.

**Figure 1:**
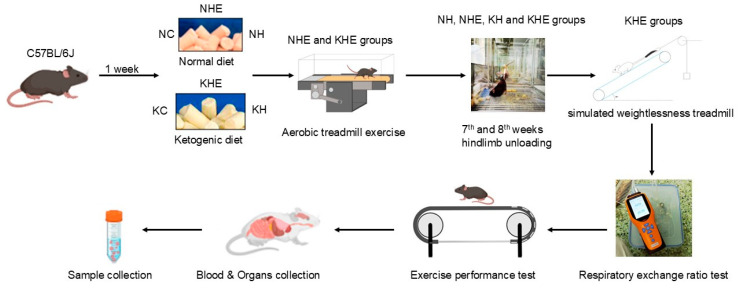
Schematic overview of experimental design.

Mice in the normal diet groups received a standard diet (AIN93G, Shanghai Puloteng Biotechnology Co., Ltd., Shanghai, China) containing 7% fat, 17.8% protein, and 64.3% carbohydrates (3.601 kcal/g). Mice in ketogenic diet groups were fed a ketogenic formula (TP-201450, Beijing BioPeak Biotechnology Co., Ltd., Beijing, China) containing 76.1% fat, 8.9% protein, and 3.5% carbohydrates (4.056 kcal/g). All animals had ad libitum access to food and water. Following a one-week acclimation period, mice were randomly allocated into experimental groups as described above. Body weight was measured weekly every Sunday between 9:00 and 10:00 AM, and blood samples were collected from tail veins for the determination of glucose and ketone concentrations using FreeStyle Optium Neo Blood Glucose/β-Ketone Test Strips (Abbott Diabetes Care Ltd., Manchester, UK). A schematic overview of the experimental design is presented in Figure 1.

### 2.2. Hindlimb Unloading Model

Simulated weightlessness was achieved through hindlimb unloading (HU) using the tail suspension method. Briefly, medical adhesive tape was applied 2–3 cm from the tip of the tail to attach it to an overhead chain, suspending the animal’s hindlimbs to avoid contact with the cage floor. The forelimbs maintained contact, allowing for free movement within the cage. Environmental conditions were maintained as described above. The hindlimb unloading intervention lasted for 2 weeks.

### 2.3. Aerobic Exercise Protocol

Aerobic exercise was conducted using a motor-driven treadmill (JD-PT, Jide Technology Co., Ltd., Beijing, China), based on the experimental protocol described by Ma et al. [14]. Mice in the exercise groups (NHE and KHE) underwent a 3-day treadmill acclimation period (5 m/min, 10 min/day, and 0° incline). Subsequently, formal training consisted of daily treadmill exercise at 12 m/min for 40 min/day, 5 days per week, over an 8-week intervention period. During the last 2 weeks, exercise intervention was carried out under simulated microgravity conditions, as described by Wang et al. [20] (Figure 1).

### 2.4. Respiratory Exchange Ratio (RER) Test

Respiratory metabolism was assessed before and after hindlimb unloading. Mice were placed individually in sealed respiratory chambers (Shenzhen Kerano Electronics Technology Co., Ltd., Shenzhen, China), and oxygen (O_2_) consumption and carbon dioxide (CO_2_) production were continuously recorded until the CO_2_ concentration reached approximately 5000 ppm. RER was calculated as VCO_2_/VO_2_.

### 2.5. Assessment of Endurance Exercise Performance

Exercise endurance capacity was evaluated using an incremental treadmill running protocol. The exercise capacity test was adapted from a study by Dougherty et al. [21], with minor modifications. Following a 1-week adaptation phase (5 m/min for 10 min/day), mice underwent incremental treadmill exercise starting at 14 m/min for 2 min, followed by stages of 16 m/min (3 min), 18 m/min (25 min), 20 m/min (15 min), 22 m/min (15 min), 24 m/min (15 min), and 26 m/min until exhaustion. Exhaustion was defined as the inability of mice to maintain pace despite repeated gentle encouragement [22].

### 2.6. Tissue Sampling

Upon completion of the final endurance exercise test, mice were anesthetized using sodium pentobarbital. Blood samples were collected via retro-orbital sinus puncture and centrifuged to obtain serum, which was stored at −20 °C. Left soleus muscles were immediately excised, weighed, snap-frozen in liquid nitrogen, and stored at −80 °C for subsequent analysis. The right soleus muscle was embedded in an optimal cutting temperature compound (OCT, Sakura, Tokyo, Japan), rapidly frozen in liquid nitrogen, and stored for subsequent sectioning and staining.

### 2.7. Plasma Biochemical Assessment

Serum levels of triglycerides (TG, A110-1-1); total cholesterol (TC, A111-1-1); high-density lipoprotein cholesterol (HDL-C, A112-1-1); low-density lipoprotein cholesterol (LDL-C, A113-1-1); insulin; creatine kinase (CK, A032-1-1); lactate dehydrogenase (LDH, A020-2-2); blood urea nitrogen (UREA, C013-2-1); insulin (A019-2-1); and blood lactate (LA, H203-1-1) were measured using an automated biochemical analyzer (Huake Bio, Beijing, China). All procedures were conducted in strict accordance with the manufacturer’s instructions provided by the reagent kits (Nanjing Jiancheng Bioengineering Institute, Nanjing, China).

### 2.8. Oil Red O Staining

The OCT-embedded tissue samples were retrieved from liquid nitrogen and sectioned to a thickness of 8 µm using a cryostat (Leica, Wetzlar, Germany). The sections were mounted onto slides, with six sections per tissue sample, and placed in a slide box. The slides were stored at −20 °C until further use. Lipid staining was performed following the instructions provided by the Oil Red O Staining Kit (G1260, Solarbio, Beijing, China). Tissue sections were initially washed in 60% isopropanol for 2 min, followed by staining with the Oil Red O working solution for 2–5 min. After staining, the sections were rapidly differentiated in 60% isopropanol and washed with distilled water. Subsequently, the sections were counterstained with hematoxylin for 30 s, differentiated with acid alcohol for 1–5 s, and rinsed under running water until a blue color was achieved. Finally, the sections were mounted with glycerol gelatin and observed under a light microscope, with images captured.

### 2.9. Periodic Acid–Schiff (PAS) Staining

The prepared frozen sections were retrieved and sequentially stained with periodic acid, Schiff’s reagent, and hematoxylin, in accordance with the instructions provided in the Periodic Acid–Schiff (PAS) Staining Kit (G1281, Solarbio, Beijing, China). Following washing and dehydration, the sections were mounted with neutral gum. The tissue sections were observed under an optical microscope, and images were captured and saved.

### 2.10. Quantitative Real-Time Polymerase Chain Reaction

A total of 10 mg of soleus muscle tissue was weighed, and 1 mL of the TRIzol reagent was added (Invitrogen, Carlsbad, CA, USA) to homogenize the sample. After separating the supernatant, 200 µL of chloroform was added and mixed thoroughly. The mixture was left to stand at room temperature for 5 min. Then, it was centrifuged at 12,000 rpm at 4 °C for 10 min to collect the aqueous phase. A total of 500 µL of isopropanol was added and mixed gently. Then, the mixture was left to stand at room temperature for 10 min. It was centrifuged again at 12,000 rpm at 4 °C for 10 min, the supernatant was discarded, and the RNA pellet was washed with 1 mL of pre-cooled 75% ethanol. The mixture was centrifuged at 7500 rpm at 4 °C for 5 min, the supernatant was removed, and the pellet was left to air-dry for 15 min to remove residual ethanol. An appropriate volume of DEPC-treated water was added to fully dissolve the RNA pellet and measure the RNA concentration. Genomic DNA was removed using the Master Mix Kit RR047A, Takara Bio USA, Mountain View, CA, USA), and RNA was reverse transcribed using the same kit. Quantitative RT-PCR was performed using TB Green Premix Ex Taq (RR420A, Takara Bio USA, Mountain View, CA, USA). The 18S rRNA gene was used as the internal reference, and relative mRNA expression was calculated using the 2^−ΔΔCt^ method. The RT-PCR primers used in this study are listed in Table 2.

### 2.11. Histological Staining

For staining, the cryosections were blocked with 3% bovine serum albumin (BSA) in DPBS for 1 h at room temperature, followed by incubation overnight at 4 °C with a cocktail of primary antibodies, including MyHC I (BA-D5, DSHB, Iowa City, IA, USA); MyHC IIa (SC-71, DSHB); MyHC IIb (BF-F3, DSHB); and Laminin (ab11575, Abcam, Cambridge, UK). After washing, the cryosections were incubated with a cocktail of secondary antibodies, including Alexa Fluor 647-conjugated goat anti-mouse IgG2b (A21242, Invitrogen, Thermo Fisher Scientific, Carlsbad, CA, USA); Alexa Fluor 568-conjugated goat anti-mouse IgG1 (A21124, Invitrogen, Thermo Fisher Scientific, Carlsbad, CA, USA); Alexa Fluor 488-conjugated goat anti-mouse IgM (A21042, Invitrogen, Thermo Fisher Scientific, Carlsbad, CA, USA); and Alexa Fluor 405-conjugated goat anti-rabbit IgG (A31556, Invitrogen, Thermo Fisher Scientific, Carlsbad, CA, USA), for 1 h at room temperature. Images of the cryosections were acquired using a Leica Thunder Imager 3D Assay (Leica Application Suite X (LAS X) 3.6.0, Leica) and analyzed with the Image J software (version 1.53t; NIH, Bethesda, MD, USA). To minimize observer bias, the researchers performing image acquisition and analysis were blinded to group assignments. Tissue sections were labeled with anonymous codes (e.g., A1–F6) prior to staining, and group identities were revealed only after statistical comparisons were completed.

### 2.12. Controlled Variables and Measurement Validation

To ensure reproducibility and transparency, all experimental variables, measurement instruments, and validation references are summarized in Table 3. Key biochemical assays followed standardized protocols provided by reagent manufacturers, while histological and molecular analyses were validated against established methodologies.

### 2.13. Statistical Analysis

Statistical analysis was performed via GraphPad Prism 10.0 (GraphPad Software, San Diego, CA, USA) and the SPSS 22.0 software (IBM, Armonk, NY, USA). The experimental results are presented as means ± standard deviations (means ± SD). After confirming a normal distribution, one-way analysis of variance (ANOVA) was employed for statistical analysis, with pairwise comparisons conducted via the least significant difference (LSD) method, and post hoc tests were performed via Duncan’s multiple range test. A value of *p* < 0.05 was considered statistically significant.

## 3. Results

### 3.1. Establishment of the Ketogenic Diet Model

**Figure 2:**
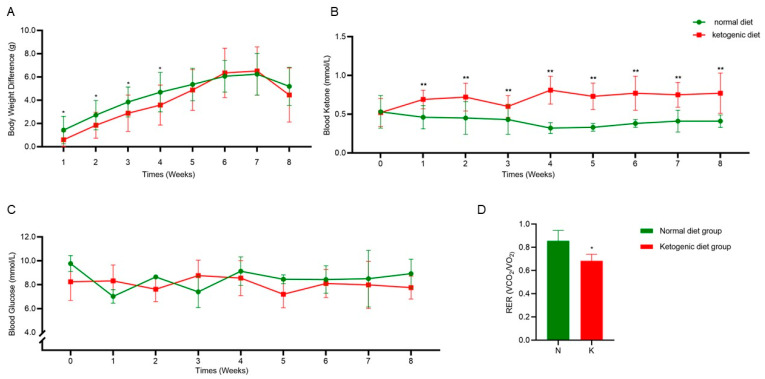
Successful establishment of the ketogenic diet in a mouse model. (**A**) Weekly changes in mouse body weight. (**B**) Weekly variations in blood ketone levels. (**C**) Weekly fluctuations in blood glucose levels. (**D**) Changes in the respiratory exchange ratio. The data are presented as means ± SD. * *p* < 0.05; ** *p* < 0.01, compared with the normal diet control group. NC: normal diet control group; KC: ketogenic diet control group; RER: respiratory exchange ratio.

As shown in Figure 2A, the weight difference among the mice from all groups gradually increased with age. During the first 4 weeks, the weight gain in the KD group was significantly lower than that in the normal control (NC) group (*p* < 0.05). However, after week 5, no significant changes in the weight difference were observed between the dietary groups. Figure 2B demonstrates that compared to the NC group, the ketone levels in the KD group were significantly higher from week 1 to week 8 (0.73 ± 0.06 mmol/L, *p* < 0.01), while blood glucose levels remained unchanged (Figure 2C). Research indicates that when ketone levels reach 0.5–3.0 mmol/L, the body is considered to be in a state of nutritional ketosis [26]. The RER, which represents the ratio of carbon dioxide produced to oxygen consumed during metabolism, reflects the balance between fat and carbohydrate metabolism. As seen in Figure 2D, the RER in the KD group was significantly lower than that in the NC group (*p* < 0.05), maintaining a value of around 0.7. Studies suggest that an RER value between 0.7 and 1.0 indicates a mixed utilization of fat and carbohydrates for energy [27]. These results suggest that a short-term ketogenic diet intervention in mice significantly reduces weight gain, increases ketone production, enhances the process of ketone body generation from fat breakdown, and lowers the RER, indicating successful establishment of the ketogenic diet in a mouse model.

### 3.2. Effects on Body Weight and Skeletal Muscle Mass in Simulated Weightlessness Mice

**Figure 3:**
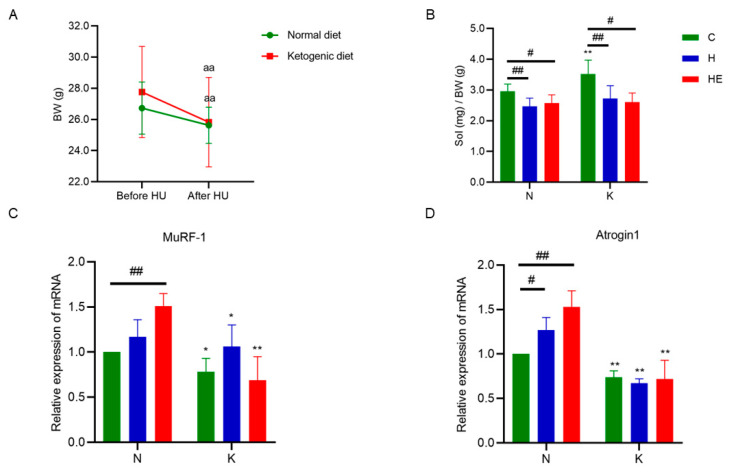
Effects of simulated weightlessness on muscle atrophy markers. (**A**) Body weight fluctuations in the mice before and after hindlimb unloading. (**B**) Ratio of soleus muscle wet weight to body weight across groups. mRNA expression of MuRF-1 (**C**) and Atrogin1 (**D**) in the soleus muscle. The data are presented as means ± SD. ^aa^ *p* < 0.01, compared with before HU; * *p* < 0.05; ** *p* < 0.01 compared with the control group; ^#^ *p* < 0.05; ^##^ *p* < 0.01, compared with the diet groups. BW: body weight; NC: normal diet control group; NH: normal diet + hindlimb unloading group; NHE: normal diet + hindlimb unloading + exercise group; KC: ketogenic diet control group; KH: ketogenic diet + hindlimb unloading group; KHE: ketogenic diet + hindlimb unloading + exercise group.

As shown in Figure 3A, both the KC and NC groups exhibited significant body weight reduction (*p* < 0.05) after two weeks of tail suspension. Compared to the NC group, the soleus muscle wet weight-to-body weight ratio decreased significantly in the NH and NHE groups (*p* < 0.05; Figure 3B). Notably, the NH group showed a marked elevation in skeletal muscle Atrogin1 mRNA expression (*p* < 0.05; Figure 3D). Previous studies have established that reduced muscle wet weight-to-body weight ratios and increased expression of skeletal muscle atrophy markers (MuRF-1 or Atrogin1) are hallmarks of muscle atrophy [28]. These results confirm the successful establishment of a weightlessness-induced skeletal muscle atrophy model. In contrast, the KH and KHE groups displayed significant reductions in the soleus muscle wet weight-to-body weight ratio compared to the KC group (*p* < 0.05), though no significant changes were observed in MuRF-1 or Atrogin1 mRNA expression (Figure 3C,D). Intergroup comparisons further revealed significant decreases in soleus muscle MuRF-1 and Atrogin1 mRNA expression between the NC and KC, NH and KH, and NKE and KHE groups (*p* < 0.05). Collectively, these findings indicate that weightlessness conditions induce muscle atrophy in both standard and ketogenic diet groups, whereas a ketogenic diet alone or combined with exercise intervention effectively counteracts this atrophy.

### 3.3. Effects on Glucose and Lipid Metabolism in Mice

**Figure 4:**
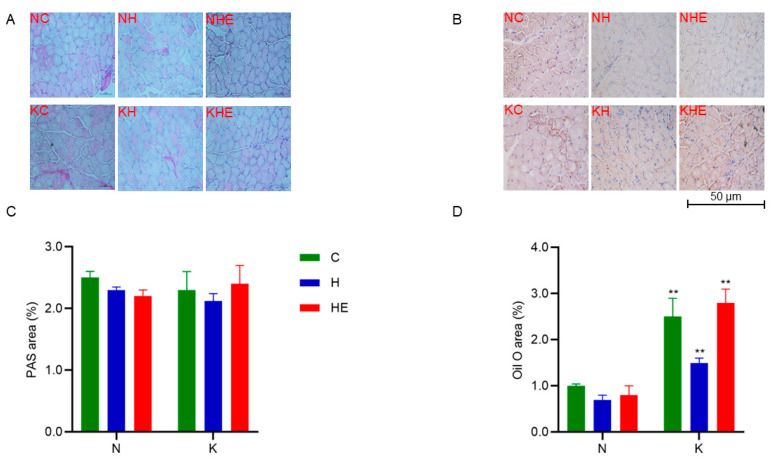
Effects of KD and KD combined with exercise on skeletal muscle tissue in mice. PAS (**A**) and Oil Red O (**B**) staining of the soleus muscle. Percentage of PAS- (**C**) and Oil Red O-stained (**D**) area in the soleus muscle. The data are presented as means ± SD. ** *p* < 0.01, compared between diet groups.

To examine changes in glycogen and lipid droplet content in skeletal muscle after a single bout of exhaustive exercise, PAS and Oil Red O staining were performed on skeletal muscle sections from the mice. PAS staining results (Figure 4A,C) show no significant changes in glycogen content across all groups. Additionally, Oil Red O staining results (Figure 4B,D) indicate that there were no significant differences in lipid droplet content within each dietary group. However, intergroup comparisons revealed that the lipid droplet content in skeletal muscle was significantly higher in the NC group compared to the KC group, the NH group compared to the KH group, and the NKE group compared to the KHE group (*p* < 0.05). To assess changes in blood ketone and glucose levels before and after exhaustive exercise, test strips were used. These results suggest that neither a regular nor a ketogenic diet, in combination with a weightless environment or aerobic exercise alone, significantly alters lipid droplet content in skeletal muscle. However, the ketogenic diet significantly increased lipid droplet content in skeletal muscle. Moreover, the increase in circulating ketone levels at rest indicates that the ketogenic diet enhances fat metabolism, elevating blood ketone concentrations to support prolonged exercise by promoting the utilization of ketones as an energy source.

To further examine changes in the metabolic levels of mice, biochemical analyses were conducted to measure blood metabolic parameters. As shown in Table 4, compared to the NC group, serum levels of TC, TG, LDL-C, and HDL-C in the NH and NHE groups showed no significant changes, but insulin levels were significantly increased (*p* < 0.05). Compared to the KC group, serum TC and HDL levels in the KH and KHE groups were significantly reduced (*p* < 0.05), and serum LDL levels in the KHE group were also significantly decreased (*p* < 0.05). Interestingly, the serum TC level in the KH group was significantly increased (*p* < 0.05). Notably, there were no significant changes in urea and insulin levels between the groups. Furthermore, intergroup comparisons revealed that compared to the NC group, both the KC and NH groups had significantly lower levels of serum TC, TG, LDL-C, HDL-C, urea, and insulin (*p* < 0.05). Additionally, compared to the NHE group, the KHE group had significantly lower insulin levels (*p* < 0.05), along with significantly higher levels of TC and HDL-C (*p* < 0.05), while the remaining parameters showed no significant differences. Therefore, simulated weightlessness did not affect serum lipid levels in mice on a regular diet. However, a ketogenic diet alone led to increased serum lipid levels, whereas a ketogenic diet combined with exercise partially mitigated the lipid increase induced by the ketogenic diet. These results suggest that simulated weightlessness impairs the blood glucose regulation ability in mice after exhaustive exercise. Furthermore, a ketogenic diet intervention alone did not significantly influence the reduction in blood glucose after exhaustive exercise in weightless mice, and the combination of exercise with a ketogenic diet did not show any improvement in this regard.

### 3.4. Effects on Skeletal Muscle Fiber-Type Composition

**Figure 5:**
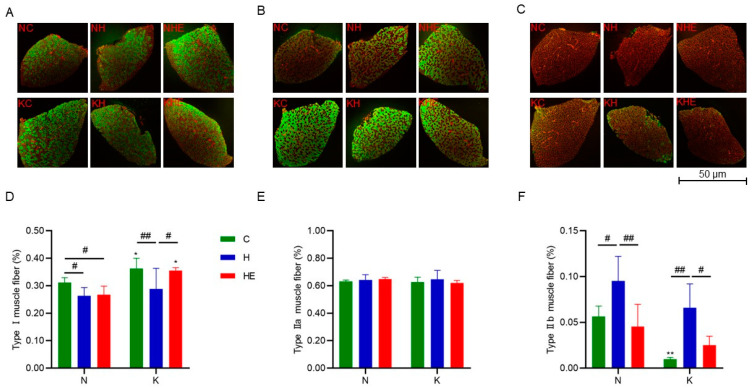
Effects of KD or KD combined with exercise on muscle fiber composition in the soleus muscles of mice. Representative immunofluorescence images of MyHC-I (blue (**A**)), MyHC-IIa (red (**B**)), and MyHC-IIb (green (**C**)) muscle fibers in the soleus muscle. The proportion of MyHC-I (**D**), MyHC-IIa (**E**), and MyHC-IIb (**F**) muscle fibers in the soleus muscle. The data are presented as means ± SD. * *p* < 0.05; ** *p* < 0.01 indicate comparisons between diet groups; ^#^ *p* < 0.05; ^##^ *p* < 0.01 indicate comparisons within each diet group.

To investigate changes in muscle fiber types, immunofluorescent staining was performed on skeletal muscle to analyze the percentage of each muscle fiber type relative to the total fiber count. As shown in Figure 5, compared to the NC group, the proportion of MyHC-I muscle fibers in the NH group was significantly reduced (*p* < 0.05), while the proportion of MyHC-IIb muscle fibers was significantly increased (*p* < 0.05). In the NHE group, the proportion of MyHC-I fibers was significantly decreased (*p* < 0.05), while no significant change was observed in the proportion of MyHC-IIb fibers. Compared to the KC group, the KH group showed a significant decrease in the proportion of MHC-I fibers (*p* < 0.05) and a significant increase in the proportion of MyHC-IIb fibers (*p* < 0.05). However, no significant changes were observed in the fiber proportions in the KHE group. Additionally, intergroup comparisons revealed that compared to the NC group, the KC group had a significantly higher proportion of MyHC-I fibers (*p* < 0.05) and a significantly lower proportion of MyHC-IIb fibers (*p* < 0.05). The proportion of MyHC-I fibers in the KHE group was significantly higher than that in the KH group (*p* < 0.05), while the decrease in MyHC-IIb fibers showed a trend but did not reach statistical significance. Notably, no significant changes in the proportion of MyHC-IIa fibers were observed across all groups. In conclusion, both regular and ketogenic diet interventions in mice under simulated weightlessness lead to a shift from slow-twitch to fast-twitch fibers in the soleus muscle. While aerobic exercise can partially suppress the increase in MyHC-IIb fibers in the soleus muscle of mice on a regular diet, it does not effectively prevent the reduction in slow-twitch fibers. However, the combination of a ketogenic diet and exercise has a significantly greater effect than exercise alone, substantially improving the muscle fiber-type transition induced by simulated weightlessness. Furthermore, under normal conditions, the ketogenic diet promotes the conversion of fast-twitch fibers to slow-twitch fibers in the soleus muscle.

### 3.5. Effects on Skeletal Muscle-Related Gene Expression

**Figure 6:**
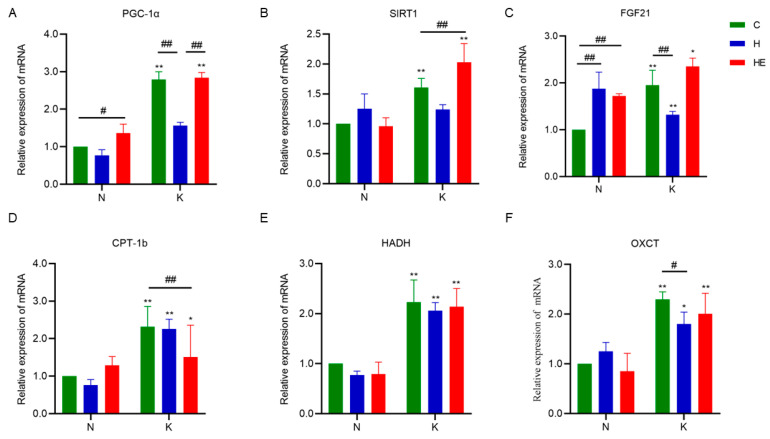
Effects of the ketogenic diet or the ketogenic diet combined with exercise on skeletal muscle mRNA expression. mRNA expression of PGC-1α (**A**), SIRT1 (**B**), FGF21 (**C**), CPT-1b (**D**), OXCT (**E**), and HADH (**F**) in the soleus muscle. The data are presented as means ± SD. * *p* < 0.05; ** *p* < 0.01, denoting comparisons between diet groups; ^#^ *p* < 0.05; ^##^ *p* < 0.01, reflecting comparisons within diet groups.

Previous studies have shown that FGF21, SIRT1, and PGC-1α are key regulators of aerobic metabolism in skeletal muscle and play pivotal roles in facilitating ketone body breakdown and fatty acid oxidation [29,30]. As shown in Figure 6A–C, compared with the NC group, the NH group exhibited significantly higher FGF21 mRNA expression in skeletal muscle (*p* < 0.05), whereas both PGC-1α and FGF21 mRNA levels were significantly upregulated in the NHE group (*p* < 0.05), with no notable changes in SIRT1 mRNA. Compared with the KC group, skeletal muscle PGC-1α and FGF21 mRNA levels were significantly decreased in the KH group (*p* < 0.05), whereas SIRT1 mRNA expression was markedly increased in the KHE group (*p* < 0.05). Furthermore, intergroup comparisons revealed that PGC-1α, SIRT1, and FGF21 mRNA expression in the KC and KHE groups were significantly higher than those in the NC and NHE groups, respectively (*p* < 0.05), whereas FGF21 mRNA in the KH group was significantly lower than that in the NH group (*p* < 0.05).

In addition, OXCT is an essential ketolytic enzyme in skeletal muscle and reflects the ketone body breakdown process [31]. CPT-1b serves as a crucial fatty acid transporter involved in β-oxidation [32,33], whereas HADH is the rate-limiting enzyme in the β-oxidation of fatty acids in skeletal muscle [34]. Changes in the expression of these enzymes can thus indicate the extent of lipid metabolism in murine skeletal muscle. As shown in Figure 6D–F, there were no significant changes in CPT-1b, HADH, or OXCT mRNA expression in the NH and NHE groups compared with the NC group. However, relative to the KC group, OXCT mRNA in the KH group was significantly reduced (*p* < 0.05), and CPT-1b mRNA in the KHE group was also markedly decreased (*p* < 0.05). Moreover, intergroup comparisons indicated that CPT-1b, HADH, and OXCT mRNA levels in the KC, KH, and KHE groups were significantly higher than those in the NC, NH, and NHE groups, respectively (*p* < 0.05).

### 3.6. Effects on Exercise Capacity in Mice

To investigate the fatigue recovery capacity of mice following a single bout of exhaustive exercise, a biochemical analyzer was used to measure blood markers associated with fatigue. As shown in Table 5, compared to the NC group, the serum LA levels in the NH and NHE groups were significantly elevated (*p* < 0.05), with no significant difference observed between the NH and NHE groups. Additionally, the serum LD levels in the KHE group were significantly lower than those in the KC group (*p* < 0.05), while no significant differences were observed for other markers. Intergroup comparisons revealed that the serum LA levels in the KC group were significantly higher than those in the NC group, suggesting that the ketogenic diet leads to increased lactate accumulation post-exercise, which may be associated with the metabolic shifts induced by the ketogenic diet.

The single exhaustive exercise test serves as a measure of exercise endurance. As shown in Table 3, compared to the NC group, the exercise exhaustion time was significantly reduced in the NH and NHE groups (*p* < 0.05). When compared to the KC group, the exercise exhaustion time was also significantly reduced in the KH and KHE groups (*p* < 0.05), although the KHE group exhibited a significantly longer exhaustion time than the KH group (*p* < 0.05). Furthermore, intergroup comparisons revealed no significant differences in exercise exhaustion times between the NC and KC groups or between the NH and KH groups. However, the exercise exhaustion time in the KHE group was significantly longer than that in the NHE group (*p* < 0.05). In conclusion, both standard and ketogenic diets led to a reduction in exercise endurance under simulated weightlessness. Neither aerobic exercise nor ketogenic diet interventions alone were able to counteract the decline in exercise endurance induced by simulated weightlessness, whereas the combination of a ketogenic diet and aerobic exercise significantly mitigated the decrease in exercise endurance in mice.

## 4. Discussion

### 4.1. Effects of Ketogenic Diet Combined with Aerobic Exercise on Body Weight and Skeletal Muscle in Simulated Weightlessness Mice

The ketogenic diet (KD), characterized by high-fat, low-carbohydrate, and moderate-protein intake, has gained considerable attention for its potential health benefits [35,36]. Previous research has demonstrated that a 4-week KD intervention effectively prevented age-related weight gain in rodents [9], consistent with other published reports [37,38,39,40,41,42,43,44]. Similarly, the present study showed that after 4 weeks of dietary intervention, C57BL/6 mice in the KD group exhibited significantly lower body weight gains compared to controls, beginning from the second week, corroborating earlier findings [45,46,47]. However, this weight difference progressively diminished with a longer intervention duration, suggesting the development of keto-adaptation, potentially explaining the phased weight changes observed. Notably, these results contrast with findings from Shimizu et al. [16], Wallace et al. [8], and Zhou et al. [48], possibly due to differences in intervention duration, dietary protein content, or animal strain.

Currently, limited research has explored the effects of KD under simulated microgravity conditions. Typically, body weight, muscle wet weight, and the muscle-to-body-weight ratio are standard parameters for evaluating muscle atrophy in rodent models, with MuRF-1 and Atrogin1 recognized as biomarkers for skeletal muscle wasting [49]. In the present study, 2 weeks of hindlimb unloading significantly decreased body weight and the soleus muscle wet weight-to-body weight ratio and elevated Atrogin1 expression in mice on a normal diet, confirming muscle atrophy induction. Notably, subsequent dietary and exercise interventions revealed that KD alone or combined with exercise significantly downregulated MuRF-1 and Atrogin1 expression in soleus muscles, mitigating muscle atrophy progression. Intriguingly, KD alone also demonstrated protective effects on soleus muscle integrity and mass under normal conditions. However, further extensive studies are required to validate these findings comprehensively.

### 4.2. Effects of Ketogenic Diet Combined with Aerobic Exercise on Skeletal Muscle Fiber Remodeling in Simulated Weightlessness Mice

Sandonà et al. [50] demonstrated that prolonged microgravity exposure results in approximately a 35% reduction in the cross-sectional area of soleus muscle fibers (anti-gravity, slow-twitch muscles) in mice, accompanied by homogeneous atrophy across all fiber types (MyHC-I, MyHC-IIa, and MyHC-IIx). Concurrently, a significant slow-to-fast fiber phenotype transition was observed, characterized by decreased proportions of MyHC-I fibers and increased proportions of fast isoforms (MyHC-IIa and MyHC-IIb). This shift may impair oxidative metabolic capacity and consequently reduce muscular endurance [51]. Consistent with these observations, our results reveal that a 2-week HU intervention significantly reduced the proportion of MyHC-I fibers while increasing MyHC-IIb fibers in mice, correlating with decreased aerobic endurance, as evidenced by exhaustive exercise tests. Previous research has indicated that KD could induce beneficial skeletal muscle remodeling by promoting the conversion of MyHC-IIb to MyHC-I fibers, potentially counteracting muscle fiber loss associated with sarcopenia [8,9,52,53,54]. Furthermore, exercise-induced reductions in MyHC-IIb isoforms have been associated with improved muscular aerobic capacity [55,56,57]. Our data support these findings, demonstrating that the combination of KD and aerobic exercise intervention produced superior outcomes compared to exercise alone, effectively ameliorating fiber-type transitions induced by simulated microgravity.

Notably, the FGF21-SIRT1-PGC-1α signaling pathway plays a critical role in promoting myocyte differentiation and facilitating anaerobic-to-oxidative fiber-type conversions [30]. Consistent with this mechanism, our study found significantly increased mRNA expression levels of PGC-1α, SIRT1, and FGF21 in the soleus muscles of mice receiving the ketogenic diet compared to regular diet controls. Immunofluorescence staining further confirmed a higher proportion of MyHC-I fibers and reduced MyHC-IIb fibers in the KD groups relative to regular diet groups. However, KD alone did not completely counteract the fiber-type shift induced by hindlimb unloading, as evidenced by the persistent slow-to-fast fiber conversion in the KH group. These pathophysiological alterations significantly impaired aerobic endurance, as demonstrated by reduced performance in exhaustive exercise tests. In contrast, the combined KD and aerobic exercise intervention yielded superior outcomes, effectively mitigating fiber-type transitions induced by simulated microgravity and significantly improving exercise performance.

### 4.3. Effects of Ketogenic Diet Combined with Aerobic Exercise on Metabolic Adaptations in Skeletal Muscle in Simulated Weightlessness Mice

Our study demonstrated that KD significantly increased lipid droplet content in skeletal muscle and enhanced systemic lipid metabolism, consistent with the findings of Ogura et al. [9] and Huang et al. [58]. KD promotes greater utilization of lipids as a fuel source, thereby reducing reliance on glucose metabolism [14,59,60]. Our experimental data show that KD effectively maintained blood glucose levels and preserved intramuscular glycogen content, suggesting improved metabolic flexibility. The observed increase in intramuscular lipid droplets provides a mechanistic explanation for the enhanced aerobic capacity observed with KD and its combination with aerobic exercise interventions. Chronic KD combined with aerobic exercise specifically promoted the remodeling of the muscle fiber composition toward aerobic fiber predominance in hindlimb-unloaded mice, optimizing metabolic efficiency by increasing lipid droplets and maintaining glycogen stores. These coordinated adaptations facilitated a metabolic shift toward enhanced lipid and ketone body utilization, significantly improving endurance performance and reducing fatigue biomarkers following exhaustive exercise.

As the mitochondrial transporter for fatty acid β-oxidation [61], CPT-1b cooperates with HADH, a key rate-limiting enzyme in this process [34]. Ketogenic diet upregulated CPT-1b and HADH expression in response to increased circulating free fatty acids and intramuscular lipid accumulation [62]. Enhanced β-oxidation elevated NAD+ levels, which activated SIRT1 expression. Subsequently, SIRT1 deacetylated and activated PGC-1α [63], forming a positive regulatory loop that further amplified lipid-metabolizing enzyme expression [64,65]. Concurrently, the FGF21-SIRT1-PGC-1α axis promoted a fast-to-slow fiber transition [30], counteracting an HU-induced fiber-type shift and establishing a slow-twitch dominant phenotype. This fiber-type remodeling synergized with persistent ketogenic intervention to augment intramuscular triglyceride storage—a recognized biomarker of aerobic capacity [58,66] that enhances endurance performance through sustained energy provision [67]. This adaptive cascade ultimately elevated CPT-1b and HADH expression in soleus muscles, collectively enhancing lipid metabolism efficiency. The ketogenic diet thus establishes a self-reinforcing cycle where improved aerobic capacity drives ketogenesis [68,69], with hepatic ketone bodies entering skeletal muscles via circulation. Notably, PGC-1α-mediated OXCT upregulation (the rate-limiting enzyme in ketolysis) facilitated ketone conversion into TCA cycle intermediates [70], completing the metabolic adaptation that significantly improved aerobic endurance. Remarkably, despite profound lipid/ketone metabolic alterations, the combined intervention maintained stable glycogen reserves and glycemic control (albeit with slight reductions), enabling sustained exercise capacity. This metabolic flexibility—characterized by ketone-dominated energy production, enhanced lipid utilization, and preserved glucose homeostasis—collectively optimized murine aerobic endurance.

## 5. Conclusions

This study demonstrated that a ketogenic diet combined with aerobic exercise effectively mitigates simulated microgravity-induced skeletal muscle atrophy and endurance impairments. The beneficial effects were mediated through the following: (1) increasing the proportion of MyHC-I fibers, (2) upregulating lipid metabolism-related genes (CPT-1b and HADH), and (3) suppressing muscle atrophy markers (MuRF-1). Future studies should incorporate larger sample sizes, utilize multi-omics approaches (e.g., metabolomics and proteomics), and perform detailed mechanistic validations. Moreover, exploring gender-specific responses and long-term efficacy will further advance translational applications in aerospace medicine, sports science, and nutritional interventions.

## Figures and Tables

**Table 1:** Experimental group assignment and sample sizes.

Diet	Intervention	Group	*n*
Normal diet (ND)	Control	NC	8
	Hindlimb unloading (HU)	NH	12
	HU + exercise	NHE	14
Ketogenic diet (KD)	Control	KC	8
	Hindlimb unloading (HU)	KH	12
	HU + exercise	KHE	14

**Table 2:** Description of primers used for quantitative real-time PCR.

Gene Name	Forward Primer	Reverse Primer
Atrogin1	5′-TCAGCAGCCTGAACTACGAC-3′	5′-GCGCTCCTTCGTACTTCCTT-3′
MURF-1	5′-GTGTGAGGTGCCTACTTGCT-3′	5′-GACTTTTCCAGCTGCTCCCT-3′
PGC-1α	5′-AGCCGTGACCACTGACAACGAG-3′	5′-GCTGCATGGTTCTGAGTGCTAAG-3′
SIRT1	5′-TACCTTGGAGCAGGTTGCAG-3′	5′-GCACCGAGGAACTACCTGAT-3′
FGF21	5′-GCATACCCCATCCCTGACTC-3′	5′-GGATCAAAGTGAGGCGATCC-3′
CPT-1b	5′-TTCAACACTACACGCATCCC-3′	5′-GCCCTCATAGAGCCAGACC-3′
HADH	5′-ACACCTTCATTCGCCATATTGC-3′	5′-TCGGTGAATTTTCTGTAGACCAC-3′
OXCT	5′-CCCATACCCACTGAAAGACGAA-3′	5′-CTGGAGAAGAAAGAGGCTCCTG-3′
18s	5′-GGGAGCCTGAGAAACGGC-3′	5′-GGGTCGGGAGTGGGTAATTT-3′

**Table 3:** Controlled variables and measurement instruments.

Variable	Measurement Instruments	Validation Reference
Body weight	Electronic Balance	Manufacturer’s protocol
Blood ketone/glucose	FreeStyle Optium Neo Blood β-Ketone/Glucose Test Strips	Abbott technical manual [23]
RER	Indirect Calorimetry	Manufacturer’s protocol
TG	The reagent kits	Manufacturer’s protocol
TC	The reagent kits	Manufacturer’s protocol
HDL-C	The reagent kits	Manufacturer’s protocol
LDL-C	The reagent kits	Manufacturer’s protocol
Insulin	The reagent kits	Manufacturer’s protocol
Glycogen content	The Periodic Acid–Schiff Staining Kit	Solarbio technical manual [24]
Lipid deposition	The Oil Red O Staining Kit	Solarbio technical manual [24]
LD	The reagent kits	Manufacturer’s protocol
UREA	The reagent kits	Manufacturer’s protocol
CK	The reagent kits	Manufacturer’s protocol
LDH	The reagent kits	Manufacturer’s protocol
Muscle fiber types	Immunofluorescence (MyHC Antibodies)	Manufacturer’s protocol [25]

**Table 4:** Plasma metabolic parameters.

	TC (mmol/L)	TG (mmol/L)	HDL-C (mmol/L)	LDL-C (mmol/L)	UREA (mmol/L)	Insulin (ng/mL)
NC	1.35 ± 0.15	0.38 ± 0.07	1.18 ± 0.09	0.17 ± 0.03	9.57 ± 0.37	0.97 ± 0.21
NH	1.29 ± 0.23	0.58 ± 0.18	1.12 ± 0.13	0.18 ± 0.06	9.28 ± 0.34	1.72 ± 0.58 ^##^
NHE	1.24 ± 0.21	0.59 ± 0.29	1.09 ± 0.21	0.20 ± 0.06	8.33 ± 0.30	1.44 ± 0.56 ^##^
KC	2.16 ± 0.20 **	0.63 ± 0.20 **	1.90 ± 0.12 **	0.27 ± 0.04 **	9.31 ± 0.80 **	1.51 ± 0.59 *
KH	1.96 ± 0.23 ^#^**	0.99 ± 0.16 ^##^**	1.64 ± 0.27 ^##^**	0.25 ± 0.03 **	9.53 ± 0.85 **	1.38 ± 0.51 **
KHE	1.79± 0.36 ^##^**	0.74 ± 0.29 ^#^	1.55 ± 0.38 ^##^**	0.23 ± 0.03 ^##^	9.25 ± 0.42 **	1.65 ± 0.71 **

Note: The data are presented as means ± SD. * *p* < 0.05; ** *p* < 0.01, compared between diet groups; ^#^ *p* < 0.05; ^##^ *p* < 0.01, compared within diet groups. TC: total cholesterol; TG: triglycerides; HDL-C: high-density lipoprotein cholesterol; LDL-C: low-density lipoprotein cholesterol.

**Table 5:** The effects of exercise capacity in mice.

	LA (mmol/L)	UREA (mmol/L)	CK (U/L)	LDH (U/L)	Time (min)
NC	1.81 ± 0.26	9.57 ± 0.37	850 ± 310	513 ± 166	136 ± 15
NH	2.41 ± 0.53 ^##^	9.27 ± 0.34	866 ± 260	655 ± 97	83 ± 24 ^##^
NHE	2.32 ± 0.34 ^##^	8.33 ± 0.30	749 ± 275	547 ± 113	84 ± 30 ^##^
KC	2.43 ± 0.39 **	9.20 ± 0.71	981 ± 233	521 ± 180	152 ± 18
KH	2.23 ± 0.46	9.26 ± 0.61	863 ± 315	615 ± 272	81 ± 36 ^##^
KHE	2.03 ± 0.34 ^##^	9.26 ± 0.49	955 ± 288	486 ± 286	136 ± 26 ^##^*

Note: Values are expressed as means ± SD. * *p* < 0.05; ** *p* < 0.01, denoting comparisons between diet groups; ^##^ *p* < 0.01, reflecting comparisons within diet groups. LA: lactate; UREA: urea nitrogen; CK: creatine kinase; LDH: lactate dehydrogenase.

## Data Availability

The authors confirm that the data supporting the findings of this study are available within the article.
